# Bacterial Infections in Sea Turtles

**DOI:** 10.3390/vetsci10050333

**Published:** 2023-05-06

**Authors:** Valentina Virginia Ebani

**Affiliations:** 1 Department of Veterinary Sciences, University of Pisa, Viale delle Piagge 2, 56124 Pisa, Italy; valentina.virginia.ebani@unipi.it; 2 Centre for Climate Change Impact, University of Pisa, Via del Borghetto 80, 56124 Pisa, Italy

**Keywords:** sea turtle, bacteria, infections, zoonosis

## Abstract

**Simple Summary:**

Sea turtles are currently endangered due to several factors, including infectious and parasitic pathogens. Opportunistic and pathogenic bacteria are largely present in the marine environment and may infect and cause diseases in sea turtles and other animals. Even though infected sea turtles are not a relevant source of infections for humans if compared to other animals, they may be involved in the epidemiology of zoonotic bacterial agents, including those that are antimicrobial resistant; therefore, the direct and indirect contact of humans with sea turtles, their products, and the environment where they live may represent a One Health threat.

**Abstract:**

Sea turtles are important for the maintenance of marine and beach ecosystems, but they are seriously endangered due to factors mainly related to human activities and climate change such as pollution, temperature increase, and predation. Infectious and parasitic diseases may contribute to reducing the number of sea turtles. Bacteria are widespread in marine environments and, depending on the species, may act as primary or opportunistic pathogens. Most of them are able to infect other animal species, including humans, in which they can cause mild or severe diseases. Therefore, direct or indirect contact of humans with sea turtles, their products, and environment where they live represent a One Health threat. Chlamydiae, Mycobacteria, and Salmonellae are known zoonotic agents able to cause mild or severe diseases in sea turtles, other animals, and humans. However, other bacteria that are potentially zoonotic, including those that are antimicrobially resistant, are involved in different pathologies of marine turtles.

## 1. Introduction

Sea turtles are currently classified into seven living species, belonging to six genera and grouped into the family Cheloniidae and Dermochelyidae. The family Cheloniidae comprises the green turtle *Chelonia mydas* (Linnaeus, 1758), loggerhead *Caretta caretta* (Linnaeus, 1758), hawksbill *Eretmochelys imbricata* (Linnaeus, 1766), Kemp’s ridley *Lepidochelys kempii* (Garman, 1880), olive ridley *Lepidochelys olivacea* (Eschscholtz, 1829)*,* and flatback *Natator depressus* (Garman, 1880). The family Dermochelyidae comprises only the leatherback *Dermochelys coriacea* (Vandelli, 1761) [1].

Sea turtles are considered as part of two distinct ecosystems: the marine system and the beach and lower dune system. Their presence in both environments is fundamental: nesting sea turtles help beaches by depositing their eggs in the sand; and eggshells and unhatched eggs left behind provide important nutrients that nourish dune vegetation such as beach grasses, which stabilize dunes and help to prevent coastal erosion. Hatchlings are an important source of food for birds, fish, and mammals. Green turtles grazing on seagrass is an important way to keep seagrass beds healthy; leatherbacks help manage the number of jellyfish in the ocean, and hawksbills help reefs by eating sponges that compete with them for space [2].

These environmental balances are currently compromised because marine turtles are seriously endangered due to factors mainly related to human activities and climate change such as pollution, temperature increase, and predation [3]. *Caretta caretta*, *D. coriacea*, and *L. olivacea* are classed as ‘Vulnerable’; *C. mydas* as ‘Endangered’; *L. kempii* and *E. imbricata* as ‘Critically Endangered’; and *N. depressus* is ‘data deficient’ according to the IUCN (International Union for Conservation of Nature and Natural Resources) [3].

Infectious and parasitic diseases have an important impact on the health status of sea turtles and, consequently, may contribute to the reduction in their number. Bacteria are widespread in marine environments and, depending on the species, may act as primary or opportunistic pathogens. Most of them are able to infect other animals, including humans, which can cause mild or severe diseases. Therefore, direct or indirect contact of humans with sea turtles, their products, and environment where they live can represent a One Health threat [4].

The aim of the present narrative review article was to focus on cases of infections in sea turtles due to the main bacterial agents, taking in account the pathogenic role of the involved bacteria for the turtles’ health and highlighting the role of these animals as source of infections for humans.

## 2. *Chlamydia* spp.

Members of the order *Chlamydiales* are obligate intracellular Gram-negative bacteria that have been under constant change in nomenclature in recent years. To date, this order includes 9 families: *Chlamydiaceae*, *Clavichlamydiaceae*, *Cribchlamydiaceae*, *Parachlamydiaceae*, *Parilichlamydiaceae*, *Piscichlamydiaceae*, *Rhabdochlamydiaceae*, *Simkaniaceae*, and *Waddliaceae.* The family *Chlamydiaceae* contains the genus *Chlamydia*, including 14 species (*C. abortus*, *C. avium*, *C. buteonis*, *C. caviae*, *C. felis*, *C. gallinacea*, *C. muridarum*, *C. pecorum*, *C. pneumoniae*, *C. poikilothermis*, *C. psittaci*, *C. serpentis*, *C. suis*, and *C. trachomatis*) and 4 *Candidatus* species. Moreover, recently the new genus *Chlamydiifrater* gen. nov. with two new species, *Chlamydiifrater phoenicopteri* sp. nov. and *Chlamydiifrater volucris* sp. nov. has been added. Several *Chlamydia*-like, also called *Chlamydia*-related, organisms, frequently found in different animal species, have been included in the eight other families [5,6].

*Chlamydia* spp. have been recognized as agents capable of infecting mammals, birds, as well as reptiles [5]. Some reports described pathological forms in chelonians due to Chlamydiae [7,8,9] as well. In particular, terrapins and tortoises usually are asymptomatic carriers of these bacteria, but, in some cases, ocular and respiratory signs and lethal systematic infection have been observed [10,11,12]. Otherwise, data about Chlamydial infections in sea turtles are very scanty.

An outbreak of severe chlamydiosis was observed and investigated in maricultured-reared green sea turtles (*C. mydas*) in a farm located in the British West Indies [13]. Affected turtles had general symptoms such as weakness, lethargy, and inability to dive. Several animals died, and post-mortem examinations showed lesions reportable to systemic infection. Liver, spleen, and heart were the organs with the most severe alterations, similar to the lesions usually observed in mammals and birds affected by chlamydiosis. An agent defined as *Chlamydia* sp. was observed in the affected organs through Macchiavello’s stain, electron microscopy, and Chlamydial antigen detection by anti-Chlamydia monoclonal antibodies, but the Chlamydial species was not identified because of the insufficient methods available in the years of the study [13]. Successively, Bodetti et al. [14] carried out further analyses on stored blocks of paraffin embedded heart from one of those green turtles: PCR and sequencing analyses on DNA extracted from the samples allowed the identification of *C. abortus*, *C. pneumoniae,* and *Neochlamydia* sp. *Chlamydia pneumoniae* resulted as 100% similar to the human genotype A. These findings highlighted that pathogens able to infect other animals may be harbored by sea turtles. In particular, *C. abortus* is an abortive agent frequently encountered in small ruminants, which is able to cause disease also in other mammals, including humans [15,16]; *C. pneumoniae* is a human pathogen, causing respiratory disease [14].

Recently, molecular techniques have allowed for the detection of Chlamydial DNA in samples collected from loggerhead sea turtles, temporarily housed for rehabilitation at the Marine Turtle Research Centre in South Italy (Portici, Italy) [17]. During this investigation, ocular–conjunctival, oropharyngeal, and nasal swabs were collected from turtles without clinical signs and submitted for molecular analyses; samples from all investigated animals resulted PCR-positive for Chlamydiaceae, but the identification of the Chlamydial species was not possible. However, the findings highlighted the spreading of Chlamydial agents in marine turtle populations. In this study, DNA of Chlamydiae was found on the mucosa of the first tract of the respiratory system and on conjunctiva similarly to the Chlamydial localization in mammals and birds [5]. 

Further surveys to investigate the presence of Chlamydiae in both asymptomatic and symptomatic sea turtles and the identification of Chlamydial species involved are pivotal to understand the possible role of these marine animals as source of infection for other animals and humans, as well as to identify new agents able to infect and cause diseases in sea turtles. In fact, in recent years, several strains identified as *Candidatus Chlamydia* sp. or *Chlamydia*-like agents, as observed in the complex taxonomy above summarized, have been found in other animal species; therefore, it is reasonable that also sea turtles could harbor new Chlamydial bacteria.

## 3. *Mycobacterium* spp.

Bacteria of the genus *Mycobacterium* are acid fast, aerobic, non-motile, and no-spore-forming bacilli. The genus includes numerous species differentiated for pathogenic properties, environmental distribution, and host affinity. *Mycobacterium* spp. are classified into two groups: the *Mycobacterium tuberculosis* complex (MTC) and the non-tuberculous mycobacteria (NTM) [18].

NTM are largely distributed in different environments; in fact, they can be isolated from water, soil, dust, and plants [18]. NTM, including known and novel species, are also present in the marine environment, as demonstrated by the infections often diagnosed in fishes and bivalves [19,20,21,22].

Reptiles can be infected by different NTM in relation to the habitat where they live. Some reports of mycobacterial infections in tortoises and terrapins have been described [23,24,25,26,27]. Sea turtles have been found to be susceptible to some NTM species as well.

*Mycobacterium chelonae* is the most frequently encountered species in these animals. It was first isolated by Friedman in 1903 from the lung of a turtle identified at that time as *Chelonia corticata* (now *Caretta caretta*) [28]. *Mycobacterium chelonae* grows optimally in marine and other aquatic environments at temperatures ranging from 22 °C to 40 °C; therefore, its finding is not surprising in sea turtles. The ability of this mycobacterium, which usually acts as opportunistic pathogen, to cause disease seems to be related to an immunodeficiency status of the turtles, as in the cases of warm-blooded animals [29]. *Mycobacterium chelonae* often penetrates through skin lesions and, on the basis of the animal immunocompetency, can induce generalized infections.

A case of mycobacteriosis by *M. chelonae* was well described by Greer et al. [29] in *L. kempii*. The animal, recovered in a rescue center in Baltimore (USA), showed epidermal appendicular and plastral lesions and a swollen left elbow joint. *Mycobacterium chelonae* was isolated from the joint and a skin nodule; in addition, after the necropsy carried out on the animals who was euthanized due to poor prognosis for recovery, the pathogen was also cultured from lungs, liver, spleen, pericardium, and kidneys.

More recently, a case of *M. chelonae* infection in a *C. caretta* has been described. The animal was found stranded alive along the Italian Adriatic coast, displaying obtundation, tachypnea, and increased respiratory effort, but it died a few hours after. The postmortem examination revealed miliary nodules in the liver, heart, stomach, gut wall, and lungs. Histological observations detected, in all nodular lesions, a small central area of necrosis with acid-fast bacilli surrounded by epithelioid cells, macrophages, and lymphocytes. Bacteriological examinations on tissue samples isolated microorganisms that, submitted to molecular analyses, showed a restriction pattern identical to *M. chelonae* [30].

*Mycobacterium marinum* was identified in a *C. caretta* in 1977. In particular, a captive-born hatchling loggerhead sea turtle with stunted growth, emaciation, and weakness died in at the National Marine Fisheries Service in Galveston, Texas (USA); on postmortem examination, the turtle presented with a granulomatous pneumonia, from which *M. marinum* was isolated [31].

*Mycobacterium haemophilum* has been detected by PCR in the spinal cord of a juvenile leatherback turtle (*D. coriacea*) that was found stranded on the Atlantic coast of Florida (USA). A disseminated granulomatous inflammation involving the lungs, liver, spleen, kidneys, small intestine, pancreas, thymus, bone marrow, and nervous system was observed in the animal [32]. Even though available data about this bacterium included its presence in fresh or chlorinated water and water supplies, *M. haemophylum* is present in the marine environment as also demonstrated by an infection case in a man who developed chronic dermal granulomata in his right arm after receiving a coral injury in Thailand [33]. *Mycobacterium haemophilum* is known as a fish pathogen, mainly zebrafish [34]. Pathologies in sea turtles due to this mycobacterial species could be underestimated because of the difficulties to cultivate and type the microorganisms that have growth requirements different from other NTM: although the normal growth temperature for mycobacteria is 35–37 °C, *M. haemophilum* prefers a lower growth temperature of 30–32 °C and requires iron supplements such as hemin or ferric ammonium citrate; its growth is visible after about 8 weeks [35].

Surveys carried out in the past allowed researchers to detect bacteria identified as *Mycobcaterium* spp. in sea turtles, but the difficulty to culture and type these agents did not always allow for a correct identification of the agents. Keymer in 1978 [36] described a case of mycobacteriosis in a *C. mydas*, but the method of bacterial identification and anatomical location of infection were not identified. Glazebrook and Campbell in 1990 [37] identified two farmed *C. mydas* with mycobacteriosis; in both cases, the infections were diagnosed on the basis of the observation of acid-fast bacilli in lung tissues. Similarly, Brock et al. in 1976 [38] observed mycobacteria in the lungs collected from three reared hatchling Pacific green sea turtles, and, on the basis of bacteriological analysis, *M. avium* was presumed to be the etiologic agent.

## 4. Enterobacteriaceae

The family Enterobacteriaceae includes Gram-negative, facultatively anaerobic, and non-spore-forming rods grouped into numerous genera and species. These bacteria are ubiquitous in nature; many species exist as free living in diverse ecological niches, both terrestrial and aquatic environments, and some are associated with animals, plants, or insects only [39]. Members of this family are localized in the intestinal tract of mammals, birds, and reptiles that excrete the bacteria with their feces. Some species, such as *Salmonella enterica*, are well-known pathogens able to cause diseases in humans and other animals characterized by clinical forms of different severity. Other Enterobacteriaceae act as opportunistic pathogens, causing infections in different anatomical region [39].

*Salmonella enterica* and *Salmonella bongori*, with their numerous serotypes, are the most important pathogens of this family and frequently associated with infections in ophidians, saurians, and chelonians who may develop disease or serve as asymptomatic carriers [40,41,42,43].

Salmonellosis has been investigated in sea turtles that often resulted as involved in the epidemiology of these pathogens. Infected marine turtles may represent a serious threat for the health of other animals, mainly humans. In fact, they may contaminate the environment of rescue centers or natural parks where they are hosted as well as beach sands where free-ranging turtles arrive to deposit their eggs.

Conversely, Salmonellae are not always detected in samples collected from investigated sea turtles [44,45,46,47]. This could mean absence of infection, but it could also be related to latent infection; in fact, reptiles excrete *Salmonella* spp. in their feces intermittently, and the bacterial load they shed increases during periods of stress [48].

A relevant issue is related to the human consumption of sea turtles’ meat and eggs. In fact, despite some national regulations restricting the capture of sea turtles, these species remain an important resource for communities worldwide [49]. Reports of ongoing sea turtle consumption (legal and illegal) have emerged from many parts of the world [50], and human cases of salmonellosis due to the consumption of meat have been reported [51,52]. In particular, serotypes Chester [51] and Muenchen [52] caused salmonellosis in persons who ate green turtles’ meat in Australia. Salmonellae have been also isolated from leatherback turtle eggs in Grenada (West Indies), where the local population usually consumes these products [53], suggesting a real risk of infection related to this feeding habit.

*Salmonella* infection can be a threat for marine turtle health too, even though few cases have been reported in the literature. Work and collaborators [54] associated *S. enterica* serotype Typhimurium with frequent cases of granulomatous nephritis in both pelagic (7%, 9/127 analyzed animals) and stranded (47%, 21/44 analyzed animals) *L. olivacea* turtles. In severely affected turtles, the kidneys were bilaterally enlarged by chronic granulomas characterized as central areas of caseous material surrounded by fibrous connective tissue, sometimes with associated fluid-filled cysts; milder cases consisted of single or few isolated lesions, where much of the kidneys were unaffected. It was not clear if the renal localization was due to septicemia or an ascendent passage from bowel to kidneys, as ureters connect directly to the intestinal lumen in sea turtles. Granulomatous nephritis is a common pathology in marine turtles [55], but cases associated with *Salmonella* spp. have been documented only in *L. olivacea* species [54].

Isolated strains were submitted to molecular analyses and resulted in belonging to a *S. Typhimurium* variant likely adapted to olive ridley turtles [54]; however, the risk of transmission of this strain to other animals, including humans, cannot be excluded.

No chelonian-specific *Salmonella* serotype is known; therefore, salmonella infection in these animals, including sea turtles, seem related to non-specific serotypes. However, *S. enterica* Choleraesuis, a serotype known as swine specific, was cultured from the mouth of one river turtle *Podocnemis expansa* and one *Podocnemis unifilis*, as well as from eggs of *P. unifilis*, on the beaches in the National Park of Araguaia, Brazil [56]. These findings, even though they regard no-marine turtles, suggest paying attention to the role of all chelonians in the spreading of all Salmonellae.

All reptilian species are known as reservoirs of Salmonellae; they often harbor these pathogens, even though they are clinically healthy. This is probably true for sea turtles; in fact, some surveys detected Salmonellae in asymptomatic marine turtles [57].

*Salmonella enterica* was cultured from the cloaca of 3/21 (14.2%) leatherback sea turtles that used the island of St. Kitts, West Indies as a nesting ground during 2011 [58]. Successively, a survey carried out on cloacal swabs collected from sea turtles in the same island found Salmonellae through cultivation and molecular methods; 1/14 *E. imbricata turtles* resulted as PCR-positive, 3/9 leatherback turtles were PCR-positive, and, from two of them, *S. enterica* Newport was also cultured; on the other hand, the serotype Montevideo was isolated from a dry sand sample [59]. Both serotypes have been also reported in terrestrial and aquatic wild animals in the Caribbean islands; therefore, an epidemiological cycle between turtles and other animals could be assumed [60,61,62]. In addition, more recently, Edwards and collaborators [63], testing sea turtles on the same island, isolated *S. typhimurium*, *S. montevideo*, *S. newport*, and the serotypes *S*. I:6,7:-:- and *S*. I:4,5,12:-:- from cloacal swabs collected from 15/57 (26.3%) nesting leatherback turtles. The finding during the same survey of Salmonella-negative *E. imbricata* and *C. mydas* is interesting; in these cases, the cloacal samples were collected from foraging turtles. The obtained results suggested that egg laying could represent a stressful event that negatively interferes with the immune system with the consequent bacteria shedding. However, the results could be also related to the environment where turtles lived. Nesting leatherback turtles had contact with the sand while foraging hard-shell turtles did not, especially male and juvenile turtles, as only mature females return to land once hatched. Therefore, the bacterial contamination of the sand could result in the transmission of S. enterica to nesting turtles who, subsequently, come ashore to nest [63].

*Salmonella enterica* was isolated from the oviductal fluid from 3/20 nesting *C. mydas* in the Ras Al-Hadd Reserve (Oman) located between the Gulf of Oman and the Arabian Sea [64].

Recently, sea turtle fecal samples were collected from 20 loggerhead and 3 green sea turtles found stranded but still alive or caught by fisherman along the Gulf of Taranto (Ionian Sea coast, Italy); one loggerhead and two green sea turtles resulted as PCR-positive for *S. enterica* [65].

Other members of the family Enterobacteriaceae have been isolated from sea turtles on the occasion of sporadic investigation on asymptomatic animals, as well as from turtles with different pathologies [66]. Among these bacteria, *Escherichia coli* was the most frequently encountered agent. It is a bacterium usually present in the intestinal tract of all animals and, in some circumstances, may induce infections with enteric diseases when enteropathogens/enteroinvasive/enterotoxic strains are involved or infections of other anatomical sites, mainly in the genito-urinary tract of mammals [67].

A recent study investigated the presence of *E. coli* O157:H7, a zoonotic enterohaemorrhagic serotype, in *C. caretta* and *C. mydas* living in the Gulf of Taranto, but no strains were isolated [65]. However, other *E. coli* strains have been associated with some lesions observed in sea turtles, such as necrotizing and granulomatous splenitis and hepatitis, fibrinous perihepatitis, fibrinous and necrotizing enteritis, fibrinous and ulcerative esophagitis, and perinephric abscesses [55].

Furthermore, *E. coli*, as well as *Enterobacter cloacae*, was frequently found in unhatched eggs and in nesting sand samples [68].

*Citrobacter* spp. are other Enterobacteriaceae members frequently isolated from sea turtles with and without lesions. *Citrobacter freundii*, together with *Enterobacter hormaechei*, was related to septic arthritis, which progressed to generalized coelomitis and death in *C. mydas* [69]; *Citrobacter* spp. were cultured from cases of cystitis, esophagitis, ulcerative stomatitis, and the granulomatous inflammation of the lungs, spleen, liver, and kidneys [55,70].

*Morganella morganii*, *Serratia marcescens*, and *Proteus* spp. were isolated from different tissues of dead *D. coriacea* turtles, which, during post-mortem examination, showed ulcerative/purulent dermatitis, granulomatous hepatitis, splenitis and pneumonia, diverticulitis, and brain hemorrhage [71].

A fatal case of necrotizing tracheitis and bronchopneumonia caused by *Serratia proteamaculans* has been recently reported in a *C. mydas* in Australia [72]. This Enterobacteriaceae is an opportunistic agent frequently isolated from soil, plants, water, and insects [73].

Moreover, several Enterobacteriaceae, such as *E. coli* and species of the genera *Klebsiella*, *Citrobacter*, *Enterobacter*, *Morganella*, *Proteus*, *Providencia*, and *Serratia*, have been cultured during surveys about bacterial microbiota in the gastrointestinal tract and also on the nasal and oral mucosal surfaces, skin, and shell [74,75,76,77,78,79].

The findings of Enterobacteriaceae are of increasing interest not only for the health status of marine turtles but mainly for the threat related to the antibiotic-resistance frequently encountered in these isolates [57,75,76,80,81,82]. In recent years, sea turtles have been used as a sentinel species for monitoring antibiotic resistance in marine environments [82]. In fact, they are considered good sentinels of this issue because of their long lifespans and tendencies to bioaccumulate pathogens and contaminants, including antimicrobials [83,84,85].

## 5. *Vibrio* spp.

Members of the genus *Vibrio*, of the family Vibrionaceae, are Gram-negative and facultatively anaerobic bacteria; they are motile by polar flagella and naturally present in marine ecosystems and estuarine waters [86]. In areas with temperate climate, the abundance of *Vibrio* is strictly related to the seasons: during the Summer, Vibrios can easily be isolated from water, suspended particulate matter, plankton, algae, sediment, fish, shellfish, and benthic marine environments; during the Winter, the number of Vibrios noticeably decreases, and they can be found overwintering in sediments [86]. The genus includes several species, most of which are pathogenic for humans. Different types of relationships between *Vibrio* spp. and host are known: a mutualistic association between *Vibrio fischeri* and fish has been identified; a symbiotic association of *V. fischeri* with sepiolid squids, chitinous shellfish, has been observed; and parasitic relationships of some Vibrios typically affecting fish, frogs, and eels are known [86].

Sea turtles have been frequently identified as carriers of *Vibrio* spp., becoming potential sources of infections for humans. *Vibrio mimicus* is a human pathogen responsible for cholera-like diarrhea, due to the fact that some strains produce the choleric toxin [87]. It has been isolated from the sand of hatching nests, as well as the cloaca and eggs of *L. olivacea* in Costa Rica [88]. The detection of this bacterial species in eggs was of particular interest because the population in Costa Rica largely consumed eggs of olive ridley turtles. However, other *Vibrio* species were found during these surveys, as well as in other investigations regarding the other sea turtle species. *Vibrio parahaemolyticus*, *V. vulnificus*, *V. fluvialis*, *V. metschnikovii*, and *V. cholerae* were frequently isolated [46,57,88,89,90].

Furthermore, *Vibrio* spp. have been isolated from lesions of sea turtles of different species. *Vibrio alginolyticus* has been frequently encountered, although it could have been overestimated if it was not identified through multiple biochemical tests and/or molecular analysis [91]. Oros and collaborators [55] cultured *V. alginolyticus* from cases of ulcerative dermatitis, broncopneumonia, ulcerative and fibrinous esophagitis, myositis, necrotizing splenitis, necrotizing enteritis, granulomatous nephritis, and granulomatous and necrotizing hepatitis. *V. alginolyticus*, largely present in marine water, usually acts as an opportunistic pathogen after its entry through traumatic injuries of the skin, carapace, oral cavity, and esophagus; lesions observed in different tissues are related to septicemia or anatomical location [55]. Integumental ulcerative disease, characterized by many lesions, mainly hemorrhagic ulcers and necrosis, in the skin of the neck, tail, axillary and inguinal regions, and also in the shell plates, was described in a *C. caretta* at the Bermuda Aquarium (Bermuda), and it was associated with *V. alginolyticus,* even though other bacteria were isolated from the lesions [92]. This species was also related to ulcerative stomatitis, obstructive rhinitis, and pneumonia in hatchling and juvenile *C. caretta* and *C. mydas* [93].

*Photobacterium damselae* (formerly *Vibrio damsela*) was considered as the agent responsible for a fatal infection with valvular endocarditis and septicemia in a *D. coriacea* found dead along the coast of Tasmania [94].

Table 1 reports the documented cases of human infections directly associated with the consumption of meat or eggs of sea turtles.

## 6. *Aeromonas* spp.

Species of the genus *Aeromonas* are Gram-negative, facultative anaerobic, and non-spore-forming bacteria. The genus, previously included in the family Vibrionaceae, has been classified as belonging to the Aeromonadaceae family [95]. *Aeromonas* bacteria are largely present in freshwater, estuarine, and marine environments. Their range of growth temperature is between 0 °C and 42 °C; therefore, these bacteria are distributed worldwide, although a mild climate usually favors their presence. Several Aeromonas species, such as *A. hydrophila*, *A. veronii*, and *A. caviae*, are responsible for human diseases, mainly gastrointestinal forms [96]; however, members of this genus have been traditionally also related to infections in reptiles, including chelonians [97], in which they usually act as opportunistic pathogens [55,98,99].

The investigations carried out in sea turtles usually detected *Aeromonas* without arriving at the species’ identification; however, when the isolates had been submitted to typing, *A. hydrophila* was the most frequently encountered species of this genus.

*Aeromonas* spp. have been associated with cases of several infections forms in different sea turtle species such as dermatitis, stomatitis, esophagitis, rhinitis, pneumonia, osteomyelitis, splenitis, hepatitis, and myositis [55,57,100,101,102]. These forms may be the beginning of septicemic forms, as demonstrated by a recent investigation on stranded *C. caretta* in South Italy that found these bacteria as the prevalent agents cultured from several organs (lung, heart, intestine, liver, spleen, kidney) and cloacal and oral swabs [81].

A relevant finding was the detection of *A. hydrophila* in severe purulent adenitis of salt glands that represented a cause of mortality in *C. caretta*. Oros and collaborators [103] carried out bacteriological examinations on stranded loggerhead turtles with evident salt gland adenitis and cultured some bacterial species; *A. hydrophila* was the most frequently isolated agent, both in pure and mixed cultures.

Bacteria of the genus *Aeromonas* have been also isolated from unhatched eggs of sea turtles; this finding could be related to passage of the bacteria from female turtles to the eggs, but aeromonas, as well as other bacteria, could also infect eggs by penetrating shell pores where they exploit interior substrates, allowing the bacteria to proliferate [104]. In fact, *Aeromonas* bacteria are often isolated from seawater and sand samples [81,105,106]; therefore, the risk of contamination of the laid eggs is very high.

## 7. *Pseudomonas* spp.

*Pseudomonas* spp. are ubiquitous Gram-negative bacteria belonging to the family Pseudomonadaceae; they inhabit soil and water as well as animal-, human-, and plant-host-associated environments [107]. *Pseudomonas aeruginosa* is the most frequently encountered species related to animal and human infections, probably because it is the most frequently researched, but other species are known. Reptiles often are asymptomatic carriers of these opportunistic pathogens [96], and different pathologies have been associated with them [108].

Different *Pseudomonas* species have been cultured from tissues and swabs collected from both asymptomatic and sick sea turtles [55,109].

*Pseudomonas aeruginosa* and *P. putrefaciens* have been cultured from bronchoalveolar lavage samples from *C. caretta* with pulmonary disease [110].

*Pseudomonas fluorescens*, *P. putrefaciens*, *P. maltophila*, *P. putida*, and *P.stutzeri* were isolated from *C. mydas* affected by fibropapillomatosis [109]. *Pseudomonas* spp., with *Burkholderia cepacia* and *Staphylococcus* spp., were found in eyes affected by keratoconjunctivitis and ulcerative keratitis [55]. In all cases, pseudomonas agents act as opportunistic pathogens, often after their entrance through traumatic injuries [55].

The role of *Pseudomonas* spp. as opportunistic pathogens also emerged in cases of mortality of hatchling turtles; these animals can be affected by several pathologies, including yolk sacculitis, dermatitis, rhinitis, stomatitis, and pneumonia, caused or complicated by bacteria present in the environment [111].

## 8. *Enterococcus* spp.

The genus *Enterococcus* includes several species of Gram-positive bacteria usually present in the intestinal tract of all animals, but they can be also found on skin and oral cavities [112]. Usually, they bring benefits to their host related to probiotic activity and bacteriocins production, but, occasionally, they act as pathogens causing infections in humans and animals, with clinical forms ranging from mild to severe symptomatology [113]. In addition, they are largely distributed in different environments; in fact, they are found in soil, water, food, sewage, and plants [112].

Enterococcal infections in turtles have not been largely investigated, but it can be supposed that the bacteria act as opportunistic pathogens also in these animals. Tsai et al. [114] described a case of an olive ridley turtle (*L. olivacea*) admitted to a sea turtle rehabilitation facility at the National Museum of Marine Biology and Aquarium in Taiwan, showing lack of appetite and bilateral elbow joint swelling; *Enterococcus faecalis* was isolated from the affected joints, and an antibiotic-sensitivity test detected resistance to several antimicrobials. Resulting from the antibiotic resistance, the turtle died after antibiotic treatment for more than one month. This case report confirmed enterococci as multi-drug resistant bacteria, as it had been largely demonstrated in other animal species [115].

*Enterococcus fecalis* seems to be the species most frequently involved in enterococcal infections of marine turtles. It was found in different organs (bladder, brain, intestines, kidneys, liver, lungs, and muscles), suggesting a septicemic form of a *C. caretta* found dead along the coast in northern–central Italy [46].

Similarly, *E. faecalis* was isolated in cases of septicemia and osteomyelitis in cold-stunned Kemp’s ridley turtles (*L. kempii*) during their rehabilitation [116] and from a deep tissue infection in a loggerhead sea turtle caused by spotted eagle ray spines [117]. Infection cases observed in marine turtles seemed related to the introduction of enterococci via gastrointestinal perforation or through wounds from environmental contamination [117]. The presence of *E. fecalis* in the seawater environment has been directly demonstrated by Meena et al. [118], and its detection in fecal samples of wild sea turtles, such as hawksbill and green turtles from the southern coast of Brazil [119], corroborated these findings.

Other enterococcal species have been found in sea turtles. *Enterococcus cloacae* was cultured from one Hawaiian green turtle (*C. mydas*) afflicted with fibropapillomas, as well as one without this pathology [109]. Furthermore, Al-Bahry and collaborators found *E. cloacae* in oviductal fluid collected from green turtles, during the egg-laying process, in Ras Al-Hadd Reserve, located between the Gulf of Oman and the Arabian Sea [76].

*Enterococcus faecalis*, *E. faecium*, and *E. hirae* were the most common species identified in the fecal samples of wild sea turtles (*C. mydas* and *E. imbricata*) [119]; the same enterococcal species were also cultured from seabirds (*S. magellanicus*, *S. trudeaui*, and *H. melanurus*), marine mammals (*B. acutorostrata*, *M. novaeangliae*, and *G. griseus*), sea lions (*Zalophus californianus*), harbor seals (*Phoca vitulina*), and northern elephant seals (*Mirounga angustirostris*), suggesting the common circulation of these bacteria in marine environments [119,120,121].

## 9. *Staphylococcus* spp. and *Streptococcus* spp.

Members of the *Staphylococcus* genus are Gram-positive coccal bacteria; they are ubiquitous microorganisms, and most of them are animal commensals that colonize niches such as skin, nares, and diverse mucosal membranes. They act as opportunistic pathogens and have been largely associated with infections in humans and other mammalians [122].

Bacteria of the genus *Streptococcus* are Gram-positive cocci characterized by different virulence factors; they are categorized on the basis of their hemolytic pattern on blood agar as α-hemolytic, β-hemolytic, or γ-hemolytic (nonhemolytic); β-hemolytic streptococci, such as *S. pyogenes*, are typically pathogenic. *Streptococcus* spp. are common opportunistic pathogens, mainly from mammals, and are associated with a variety of diseases affecting multiple organ systems [123].

*Staphylococcus* spp. can be found as normal members of the bacterial flora of sea turtles, similarly to what has been observed in other reptiles, birds, and mammals [124,125]. *Staphylococcus* spp. were cultured from nasal and cloacal swabs collected from green turtles [126] and olive ridley turtles [127]. Different staphylococcal species, including *S. aureus* and *S. epidermidis*, have been found as components of the normal conjunctival flora of marine turtles [128], but they may be also involved in cases of keratoconjunctivitis [55].

*Staphylococcus* spp. were isolated also from cases of dermatitis, pneumonia, pleuritis, pericarditis, splenitis, nephritis, and myositis [55], and *S. lentus* has been associated with ulcerative stomatitis in green turtles [70]. Recently, *S. haemolyticus* was isolated from eggs of *C. mydas* that failed to hatch and relative nesting sites [129].

*Staphylococcus* spp. and *Streptococcus* spp. may be simultaneously involved in infections of sea turtles; in fact, they have been isolated from lesions of the digestive tract of these animals. Esophagitis, gastritis, enteritis, and hepatitis were associated with these bacteria, even though other Gram-positive and Gram-negative bacteria were cultured [44]. *Streptococcus* spp. were cultured from shoulders affected by chronic inflammation of a stranded *C. mydas* [130] and from wounds of olive ridley sea turtles [131].

Recently, *Streptococcus iniae* was isolated from tissues (kidney, liver, heart, spleen) of three dead *N. depressus*. Turtles were in an autolysis status when they were found; therefore, no gross or microscopic pathological abnormalities were observed [132]. *Streptococcus iniae* is an important aquatic pathogen responsible for disease outbreaks in freshwater and marine fishes, although disease is primarily reported in aquaculture; it is considered a zoonotic agent and has also been isolated from other aquatic animals, such as dolphins (*Inia geoffrensis* and *Tursiops truncates*), bullfrogs (*Lithobates catesbeianus*), and olive sea snakes (*Aipysurus laevis*) [132,133].

## 10. *Bacillus* spp.

The genus *Bacillus* includes several species of Gram-positive, rod-shaped, aerobic or facultatively anaerobic, spore-forming bacteria. *Bacillus* spp. are largely distributed in different environments being present in the soil, water, and other ecological niches [134].

*Bacillus* spp. have been often isolated from different samples collected from sea turtles. These bacteria were dominant in feces and the nasal cavities of investigated turtle species, such as *C. mydas*, *L. olivacea*, and *D. coriacea* [126,127,135]. *Bacillus* spp. may act as opportunistic pathogens in marine turtles, as suggested by their findings in cases of enteritis, splenitis, and pneumonia [44,55].

Different species of the genus *Bacillus*, including *B. anthracis*, *B. subtilis*, and *B. cereus*, which are well-known human pathogens, were also frequently found in unhatched eggs and sand from nesting sites [136,137].

## 11. Other Bacterial Agents

Occasionally, infections due to other bacterial agents, unusually for sea turtles in some cases, have been reported in the literature. The first case of listeriosis in a sea turtle has been recently reported. An adult female *C. caretta* stranded alive along the coast of the Abruzzo region (Italy) in Summer 2021 died after some days due to respiratory failure. The necropsy showed widespread organ lesions, mainly necrosis in many organs, gastrointestinal erosions, pericarditis, and granulomatous pneumonia, and *Listeria monocytogenes*, an agent responsible for severe disease in humans and other mammals, was isolated from the involved organs [138].

Carapacial ulcers have been associated with *Psychrobacter* sp. and Coriobacteriaceae spp., which seem to act as opportunistic pathogens; in fact, they can be isolated also from healthy green sea turtles and seawater samples [139]. However, these bacteria pose a potential risk of human infection too; *Psychrobacter* spp. may infect humans after surgery, blood transfusion, or exposure to the marine environment [140,141,142]; members of Coriobacteriaceae family, even though they are normal dwellers of mammalian body habitats such as the oral cavity and gastrointestinal and genital tracts, may cause bacteremia, periodontitis, and vaginosis [143].

Cardiobacteriaceae agents were found in carapacial ulcers of sea turtles, although they seem to have a simple role as contaminant [139]. However, bacteria of this family, also found in healthy penguins, dolphins, and whales, may cause endocarditis and wound infections in humans [144].

*Lactococcus garvieae* is a fish pathogen, also isolated from kidney and liver of a *C. caretta* [46]. *Chryseobacterium meningosepticum* has been cultured from *C. caretta* with myositis [55] and organs (lung, liver, kidney, heart, skin) of dead captive posthatchling leatherback turtles [145]. Both agents are potentially zoonotic, even if cases of infection in humans are rare [146,147].

A case of floppy flipper in a *C. mydas* has been associated with *Clostridium botulinum* [148], a zoonotic agent responsible for botulism [149]. The floppy flipper is an important clinical change associated with progressive paresis and paralysis of the flippers in sea turtles and causes death as paresis and paralysis extend to the neck, and animals are unable to lift their heads to breathe [148].

*Brucella* spp., including *B. anthropi* (formerly *Ochrobacterium anthropi*), have been isolated from nest and eggs of sea turtles [136,150,151]. The role of these pathogens in the failure of hatchings has been assumed because they were found in the eggs but not in the sand external to the nest; in addition, this finding suggests that brucellae derived from the mothers [151]. The zoonotic risk related to brucellae found in marine environments has not been clarified, but not excluded [152].

Table 2 summarizes the bacterial agents detected in unhatched eggs, hatchling turtles, and sand sampled from sea turtle nesting beaches.

## 12. Conclusions

**Figure 1:**
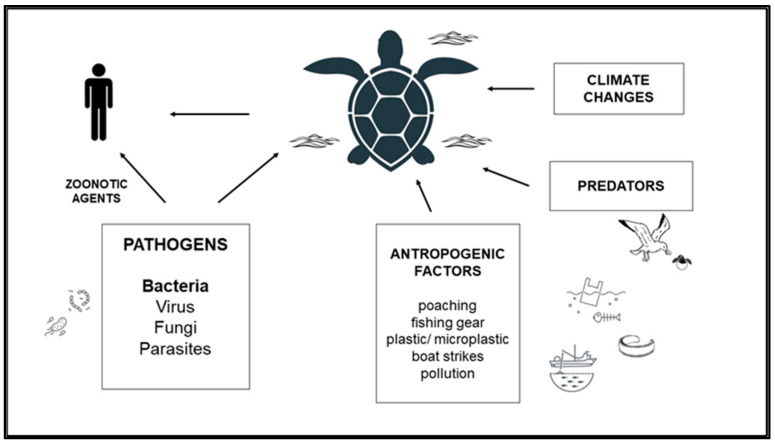
Main threats to sea turtles.

Sea turtle populations are threatened by several negative factors. Entanglement/fishing interaction, boat strike, plastic ingestion, coastal development affecting critical turtle habitat, marine pollution by inorganic elements, and organic contaminants are known as the main anthropogenic causes [71]. Moreover, there is a market for sea turtle products; the demand for sea turtle leather and shell products continues to exist despite the species’ protection under the Endangered Species Act and IUCN Red Listing. The hawksbill sea turtle (currently listed as critically endangered) is especially threatened by the illegal wildlife trade. Poachers kill the turtles for their shells and sell them as jewelry or souvenirs [153] (Figure 1).

Several pathogens may affect sea turtles causing pathologies that may be lethal in some cases. In fact, pathogens can directly cause fatal diseases or weaken turtles, which more easily may be victims of predators or injuries. Furthermore, pathogens may cause the death of embryos and hatching turtles. Among pathogens, bacteria often represent a severe threat not only for marine turtles’ health, but also for humans that come into contact with them, their products, and the environments where they live [4,154]. Sand contaminated by bacteria excreted with feces, eggs, and oviductal exudate represents a source of infection for people, mainly ecotourists, during their activity on the beach. Personnel working in rescue centers and sea turtle farms may be at risk of infection, as well.

The consumption of meat, eggs, and other products from infected sea turtles represents the major threat from a One Health perspective. Some coastal communities, especially in Central America and Asia, consume sea turtles, utilizing the entire animal. Turtle meat is eaten directly, whereas internal organs such as the kidneys and liver are used for soup [49], and the blood is drunk raw as a remedy for anemia. In addition, fat is used for extraction of oil employed as a cure for respiratory problems, especially in children [155,156]. Additionally, eggs, also valued as an aphrodisiac, are largely consumed [50]. *Salmonella* spp. and *Vibrio* spp. are considered the major bacterial hazards related to the consumption of meat and eggs, but other bacteria harbored by sea turtles may cause infection in humans through direct or indirect contact.

Bacteria harbored by sea turtles are frequently characterized by resistance to several antimicrobials; their presence in marine animals acts as an indicator of pollution levels in the surrounding area, but they also show that these animals are involved in their spreading [82]. Furthermore, antibiotic-resistant bacteria, of both human and animal origin, harbor transmissible antibiotic resistance genes. In the natural environments, including the marine one, the transfer of genetic determinants can occur [157]; therefore, new bacterial strains, pathogenetic and opportunistic, may become resistant to one or more antimicrobials.

Antibiotic-resistant bacteria should be taken into particular account as a major health concern of sea turtles because antibiotic treatment of hospitalized turtles may be unsuccessful [102,110,114,116,130,158]. However, evolution and severity of the infections, also those caused by opportunistic pathogens, are strongly related to sea turtles’ immune system status; the immune function is sensitive to fluctuations in temperature and to pollutants in the environment; and rapid changes in the marine environment are considered responsible for higher vulnerability to infections and diseases [159].

In conclusion, different pathogenic and opportunistic bacteria are able to cause infections and diseases in marine turtles. Even though infected sea turtles are not a relevant source of infections for humans if compared to other animals, they may be involved in the epidemiology of zoonotic bacterial agents, including those that are antimicrobially resistant.

## Figures and Tables

**Table 1:** Documented cases of human infections associated with sea turtles.

Pathogen	Sea Turtle Species	Origin of Infection	Country	References
*Salmonella enterica* serotype Chester	*Chelonia mydas*	Ingestion of turtle meat	Australia	[51]
*Salmonella enterica* serotype Munchen	*Chelonia mydas*	Ingestion of turtle meat	Australia	[52]
*Vibrio mimicus*	*Lepidochelys olivacea*	Ingestions of turtle eggs	Costa Rica	[86]

**Table 2:** Bacterial pathogens isolated from sea turtle eggs, hatchling turtles, and nest sand.

Bacterial Pathogen	Sample	Sea Turtle Species	References
*Aeromonas* spp.	unhatched eggs	*Dermochelys coriacea*	[104]
	nest sand	*Caretta caretta*	[81]
*Bacillus* spp.	unhatched eggs	*Chelonia mydas*	[136]
	nest sand	*Chelonia mydas*, *Eretmochelys imbricata*	[137]
*Brucella* spp.	unhatched eggs	*Chelonia mydas* *Caretta caretta*	[136] [150,151]
*Chryseobacterium meningosepticum*	hatchling turtles	*Caretta caretta*	[145]
Enterobacteriaceae (no Salmonella)	unhatched eggs, nest sand	*Lepidochelys olivacea*	[68]
*Pseudomonas* spp.	hatchling turtles	*Eretmochelys imbricata*	[111]
*Salmonella enterica* serotype Montevideo	nest sand	*Dermochelys coriacea*	[59]
*Staphylococcus* spp.	unhatched eggs	*Chelonia mydas*	[129]
*Vibrio* spp.	unhatched eggs, nest sand	*Lepidochelys olivacea*	[88]

## Data Availability

Not applicable.
